# The Cellular Basis for Biocide-Induced Fluorescein Hyperfluorescence in Mammalian Cell Culture

**DOI:** 10.1371/journal.pone.0084427

**Published:** 2014-01-28

**Authors:** May M. Bakkar, Luke Hardaker, Peter March, Philip B. Morgan, Carole Maldonado-Codina, Curtis B. Dobson

**Affiliations:** 1 Eurolens Research, Faculty of Life Sciences, The University of Manchester, Manchester, United Kingdom; 2 Medical Device Biology Group, Faculty of Life Sciences, The University of Manchester, Manchester, United Kingdom; 3 Faculty of Life Sciences, The University of Manchester, Manchester, United Kingdom; UC Berkeley, United States of America

## Abstract

Clinical examination of the ocular surface is commonly carried out after application of sodium fluorescein in both veterinary and medical practice by assessing the resulting ‘staining’. Although localized intensely stained regions of the cornea frequently occur after exposure to ‘adverse’ clinical stimuli, the cell biology underlying this staining is unknown, including whether intense fluorescein staining indicates the presence of damaged cells. Ocular exposure to certain contact lens multipurpose solutions (MPS) gives rise to intense fluorescein staining referred to as solution induced corneal staining (SICS), and we have made use of this phenomenon with Vero and L929 cell culture models to investigate the fundamental biology of fluorescein interactions with cells.

We found that all cells take up fluorescein, however a sub-population internalize much higher levels, giving rise to brightly staining ‘hyperfluorescent’ cells within the treated cultures, which contain fluorescein throughout the cell cytoplasm and nucleus. The numbers of these hyperfluorescent cells are significantly increased after exposure to MPS associated with SICS. Surprisingly, hyperfluorescent cells did not show higher levels of staining with propidium iodide, a marker of lysed cells. Consistently, treatment with the cytolytic toxin benzalkonium chloride resulted in almost all cells staining with propidium iodide, and the complete abolition of fluorescein hyperfluorescence. Finally we found that internalization of fluorescein and its loss from treated cells both require cellular activity, as both processes were halted after incubation at 4°C.

We conclude that fluorescein hyperfluorescence can be replicated in three diverse cell cultures, and is increased by MPS-treatment, as occurs clinically. The process involves the concentration of fluorescein by a sub-population of cells that are active, and does not occur in lysed cells. Our data suggest that corneal staining in the clinic reflects active living cells, and is not directly caused by dead cells being produced in response to adverse clinical stimuli.

## Introduction

The ocular surface can be affected by a number of external factors which can be examined clinically using a topical application of sodium fluorescein (‘fluorescein’) which may reveal characteristic patterns of fluorescein-associated hyperfluorescence (commonly referred to as ‘staining’) which are generally assumed to indicate deleterious cellular and/or tissue change.

Corneal staining can be readily observed at the ocular surface after exposure to toxic agents. For example, Horsley and Kahook reported reduced corneal staining when patients were switched from glaucoma eye drops containing benzalkonium chloride (a common preservative in ocular medications) to a similar drug not containing this agent [1]. Topical anesthetic drops are also associated with corneal staining [2]. Furthermore, fluorescein is routinely used to investigate and diagnose chemical injury to the cornea [3], particularly alkali burns [4]. Corneal staining can also be observed following: mechanical insult [5], ocular surface dehydration [6], [7], specific physical contact lens interactions [8], ocular surface disease and exposure to contact lens solutions [9], [10].

Few studies have addressed the precise mechanisms underpinning the interaction of fluorescein with corneal tissue. Possible aetiologies include: movement of fluorescein into the extra-cellular spaces of the corneal epithelium; uptake by epithelial cells; pooling of fluorescein in gaps or depressions at the epithelial surface; and attachment to the surface of the corneal epithelium [10].

Several studies have provided indirect evidence for transport of fluorescein into Caco-2 cell cultures [11], [12]. Moreover recent work with human corneas suggests that ‘punctate’ staining (i.e. numerous, discrete dots of fluorescence across the corneal surface) seen in dry eye is due to the localization of fluorescein within superficial epithelial cells [13]. Indeed, this work supports earlier findings of fluorescein entering rabbit corneal epithelial cells in cases of both local mechanical damage and toxic changes after the application of benzalkonium chloride [14]. However, both the subcellular location of fluorescein and whether fluorescein entry into cells occurs in all forms of corneal staining remain unknown. Furthermore, it is unclear if fluorescein staining indicates the presence of living, dead or damaged cells. Also, the temperature and time dependency of fluorescein entry into cells are unknown.

‘Solution induced corneal staining (SICS)’ is a discrete type of response, which is commonly observed after the application of soft contact lenses that have been stored in certain biocide-containing multipurpose disinfecting solutions (MPS) [9], [15], [16]. Although SICS has been described as a toxic response, it is not clear that there is damage caused to the cornea when such an appearance is observed. Its cellular basis has been assumed to involve entry of fluorescein at higher levels in damaged cells, due to increased accessibility due to a lysed cell membrane. However, the aetiology of SICS has yet to be clarified, and there at least two reports which indicate that MPS which are associated with SICS do not cause significant histological changes [17], [18].

In this work we have used epithelial and fibroblast cell culture models to investigate the fundamental cell biological mechanisms responsible for hyperfluorescence in non-confluent cells to aid our understanding of this biocide-induced clinical phenomenon.

## Materials and Methods

### Cell culture

Stock murine fibroblast (L929) cells (ECACC, Salisbury, U.K.) or African green monkey kidney epithelial (Vero) cells (ECACC, Salisbury, U.K.) were maintained in Eagle's minimum essential medium with Earle's salts, supplemented with 10% (v/v) fetal bovine serum, 4 mM L-glutamine and 1% (v/v) non-essential amino acids, 100 U/ml penicillin and 100 µg/ml streptomycin (Sigma-Aldrich Company Ltd, Gillingham, U.K.), hereafter referred to as ‘growth medium’. Telomerase-immortalized human corneal epithelial (hTCEpi) cells (obtained from the Southwestern Medical Centre, Dallas, Texas) were maintained in Clonetics KGM-2 Keratinocyte Growth Media (KGM-2) containing: KBM-2 Basal Medium, KGM-2 BulletKit, KGM-2 SingleQuot Kit Supplements and Growth Factors (all purchased from Lonza Group Ltd, Basel, Switzerland),, 20 U/ml penicillin, 20 µg/ml streptomycin, 0.5 µg/ml fungizone, (BioWhittaker, Walkersville, MD, USA), hereafter referred to as ‘hCTEpi growth medium’. Cells were incubated in a humidified incubator at 37°C and 5% CO_2_. To harvest, cells were washed with phosphate-buffered saline A (PBS) (Oxoid, Basingstoke, U.K.) before treatment with 0.25% trypsin-EDTA solution (Sigma-Aldrich Company Ltd, Gillingham, U.K.); on resuspension hTCEpi cells were treated with an equal volume of 0.25 mg/ml Soybean Trypsin Inhibitor (Gibco, Invitrogen – Life Technologies, Paisley, U.K.) After centrifugation and resuspension, cell numbers were assessed using a haemocytometer (Hausser Scientific, Horsham, PA, USA), before use in experiments.

### Cell Treatment with MPS or benzalkonium chloride

Cells were seeded into costar 24-well cell culture plates (Corning, NY, USA) for high content analysis at a density of 80,000 cells/well, in 35 mm treated cell culture dishes (Corning, NY, USA.) for confocal imaging at a density of 180,000 cells/well, or costar 96-well plate (Corning, NY, USA) at 25,000 cells/well for widefield fluorescence microscopy. The cells were cultured in a growth medium for 24 hr at 37°C. Cells were then exposed to 1∶3 dilution of PBS in growth medium (as a negative control) or 1∶3 dilution of ReNu MultiPlus® MPS (Bausch+Lomb, Kingston upon Thames, U.K.) in growth medium. The plates were then incubated for a further 15 hr at 37°C, in 5% CO_2_ in a humidified incubator prior to staining with fluorescent probes and microscopic observation. As a positive control cells were treated with a concentration of 0.01% (w/v) benzalkonium chloride (1 ml per well) (BKC; Sigma-Aldrich, Dorset U.K.) for 10 min.

### Treatment with fluorescein and fluorescent markers

Growth media were discarded from cells, and 1 ml of PBS added prior to treatment with fluorescent markers. To enable total cell numbers to be counted Hoechst 33342 (Invitrogen – Life Technologies Ltd, Paisley, U.K.) was added at a final working concentration of 41 µM and incubated for 5 min. Sodium fluorescein (Sigma-Aldrich, Dorset, U.K.) was filter sterilized using a 0.45 µm filter and added at a final concentration of 29 µM and incubated for 10 min at 37°C. This was found to be the optimal conditions for removing fluorescein associated with tissue culture plastic in preliminary experiments. The aspiration of fluorescein-containing PBS was then performed, and addition and subsequent aspiration of 2×1 ml PBS at 4°C. A final aliquot of 1 ml PBS at 4°C was then added, prior to examination (we avoided examination with cells in growth media, as this led to fading of the cellular fluorescein signal). In some experiments, Propidium iodide (PI) (Sigma-Aldrich, Dorset, U.K.) was added at a working concentration of 28 nM to visualize lysed cells.

Vybrant ® DiI (Invitrogen – Life Technologies, Paisley, U.K.) staining was used to allow identification of cell membrane. Draq5 (BioStatus Ltd, Shepshed, U.K.) was used as an alternative nuclear stain during confocal imaging due to laser availability. These dyes were used on cells seeded in 35 mm cell culture dishes. Vybrant Dil, Draq5 and fluorescein were all applied individually to cells in separate dishes, with overlapping incubation times. Growth media was discarded from the dishes and 2.5 ml of PBS added. After aspiration of different treatment solutions, 2.5 ml of PBS was added prior to treatment with fluorescent markers. Vybrant ® Dil was added at a final working concentration of 6 µM and was incubated for a total of 30 minutes; Draq5 was added at a final working concentration of 10 µM and incubated for a total of 15 minutes; sodium fluorescein (working concentration stated above) was incubated for 10 min incubation at 37°C. The aspiration of fluorescent marker containing PBS was then performed, and addition and subsequent aspiration of 2×2.5 ml PBS at 4°C. A final aliquot of 2.5 ml PBS at 4°C was then added to dishes, prior to examination via confocal microscopy. Dishes were also placed on ice during imaging to prevent diminishing fluorescein fluorescence

### High content analysis (HCA)

Quantification of cell staining was carried out using ArrayScan®II (ThermoFisher Scientific Cellomics Inc., Pittsburgh, PA, USA) with Cellomics propriety software (version 3.5.1.2). Images were collected using a 10× objective lens, a XF53 filter set and a 12-bit CCD camera, which produced a 1024×1024 format. The Valid object count feature was used then used to determine the number of cells via the Hoechst 33342 dye, and %HighCircSpotAverage area feature on the Cellomics software were used to set thresholds to determine fluorescence on intensity and size. Multiple fields of view per well were taken in each experiment.

### Fluorescence and Confocal Microscopy

Widefield fluorescence microscopy images of hTCEpi cells were collected on a Nikon Inverted TE2000i (Nikon Corporation, Tokyo, Japan) microscope using a 20×/0.50 Plan FLN objective, Cascade II camera (Photometrics, Tuscon, AZ, USA) and controlled using NIS-elements software (Nikon Corporation, Tokyo, Japan). Confocal images were collected on a Leica TCS SP5 AOBS (Leica Microsystems, Wetzlar, Germany) upright confocal using a 63×/0.90 water-dipping objective and 3× zoom. The confocal settings were as follows, pinhole 1 airy unit, scan speed 400 Hz unidirectional. Images were acquired using sequential scanning and appropriate laser and filter settings for each channel to ensure that there was no bleed through between fluorescent channels. The images were collected sequentially to eliminate cross-talk between channels. An optical section was acquired every 1 µm. Images were processed with Image J software or Leica Application Suite Advanced Fluorescence software (version 3.1.0) (Leica Microsystems, Wetzlar, Germany). Only the orthogonal views of these 3D stacks are shown in the results.

### Temperature Experiments: Mechanism of fluorescein uptake and release at 4°C and 37°C

For temperature uptake experiments, cells were bathed in PBS at 37°C or 4°C during staining, and then washed three times with PBS at 4°C (as above). For temperature release experiments, cells were bathed in PBS at 37°C during staining, and washed three times in PBS at 37°C or 4°C. Cells were monitored over 90 minutes and incubated at these temperatures between readings.

### Statistical Analysis

Data are displayed as mean ± standard error as specified. IBM SPSS version 19 statistical software (SPSS Inc, 2010) was used for unpaired t-tests and ANOVA testing, as required. The level of statistical significance was set to p = 0.05.

## Results

### Fluorescein associates with cultured L929 and Vero Cells

**Figure 1:**
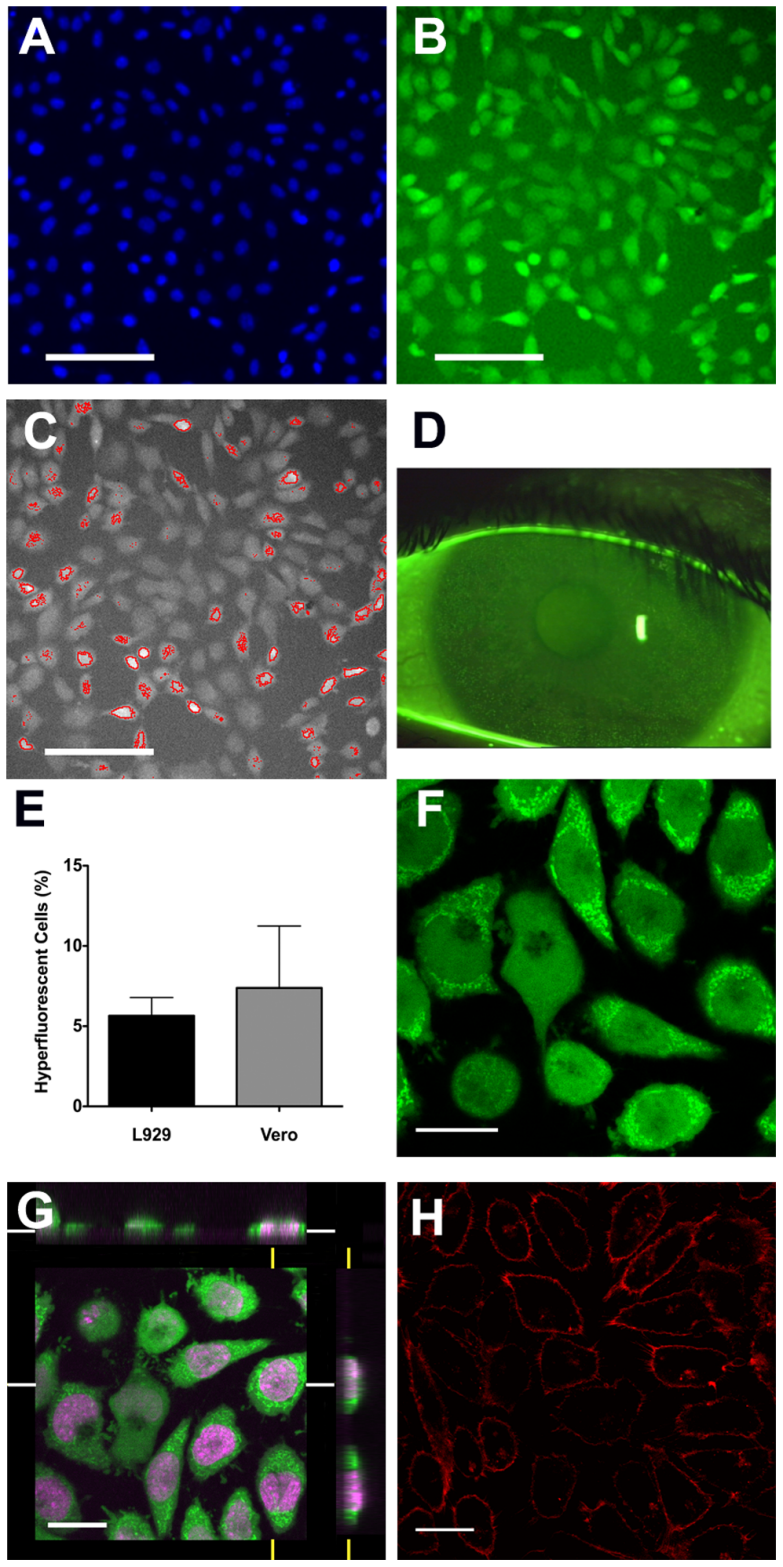
Fluorescein staining of control cell populations. L929 cell cultures were treated with fluorescein and Hoescht 33342 prior to observation. Nuclear staining is shown in (A) and fluorescein staining in (B) with typical hyperfluorecent cells visible. Images were obtained using the ArrayScan®II system and cells categorized by fluorescein intensity with hyperfluorescent cells identified shown in (C). ‘Solution induced corneal staining’, as seen on a slit lamp biomicroscope, is shown (D) for comparison, in which characteristic hyperfluorescent punctate spots are readily apparent. The proportion of hyperfluorescent cells in L929 (n = 20) and Vero cultures (n = 6) was similar (E), with bars showing standard error. Confocal microscopic analysis of Draq5 (a nuclear stain) and fluorescein-stained L929 cells, revealed the presence of fluorescein throughout the interior of the cell, with numerous highly intense fluorescein-containing structures being visible in the cytoplasm, especially of hyperfluorescent cells (F shows a single confocal ‘slice’ through the cells, and (G) shows the orthogonal view; a 3D reconstruction of staining along the white and yellow axes). Treating control cells with the membrane-slective stain Vybrant ® DiI confirmed the likely appearance after staining with a compound, which localizes on the cell surface, providing further confirmation that fluorescein has entered cells (H). Data shown are representative of several experiments. Scale bars in (A) to (C) represent 100 µm and in (F) to (H) represent 20 µm.

In initial studies we adjusted the washing conditions for fluorescein-treated L929 and Vero cell cultures to enable fluorescein associated with the cells to be clearly distinguishable from fluorescein remaining in the washes, or from fluorescein associated with regions of tissue culture flask polystyrene (to which cells had not adhered). Fluorescein was found to associate with all cells; however a sub-population of cells (around 5–10% of the total) showed elevated levels of staining which we termed ‘hyperfluorescence’ (Fig. 1A and 1B). The hyperfluorescent cells were quantified using a predetermined customized algorithm (which automatically distinguished such cells based on an assessment of cell size and fluorescein intensity over a threshold of intensity) (Fig. 1C). The differential staining pattern apparent in our cell cultures was found to be comparable in appearance to that of the human cornea after fluorescein staining, including the presence of punctate spots (potentially reflecting individual hyperfluorescent epithelial cells) (Fig. 1D). The proportion of hyperfluorescent cells in untreated/control epithelial (Vero) and fibroblast (L929) cultures was similar (7.4±3.9 and 5.7±1.1, respectively; t = 0.4, p = 0.68) (Fig. 1E).

### Subcellular localization of fluorescein association in cultured cells

Confocal microscopy revealed that fluorescein was distributed throughout the cell with levels in the cytoplasm appearing higher than those in the nucleus. In addition, there were numerous, discrete high intensity regions of fluorescein present within the cytoplasm (Fig. 1F and Fig. 1G). Comparison of the fluorescein-staining with that for a membrane-selective stain (Vybrant DiI) indicated a markedly different appearance (Fig. 1F and Fig. 1H), confirming that fluorescein does not simply adhere to cell surfaces without being internalized.

### Evaluation of cell hyperfluorescence following exposure to MPS in Vero and L929 cell lines

**Figure 2:**
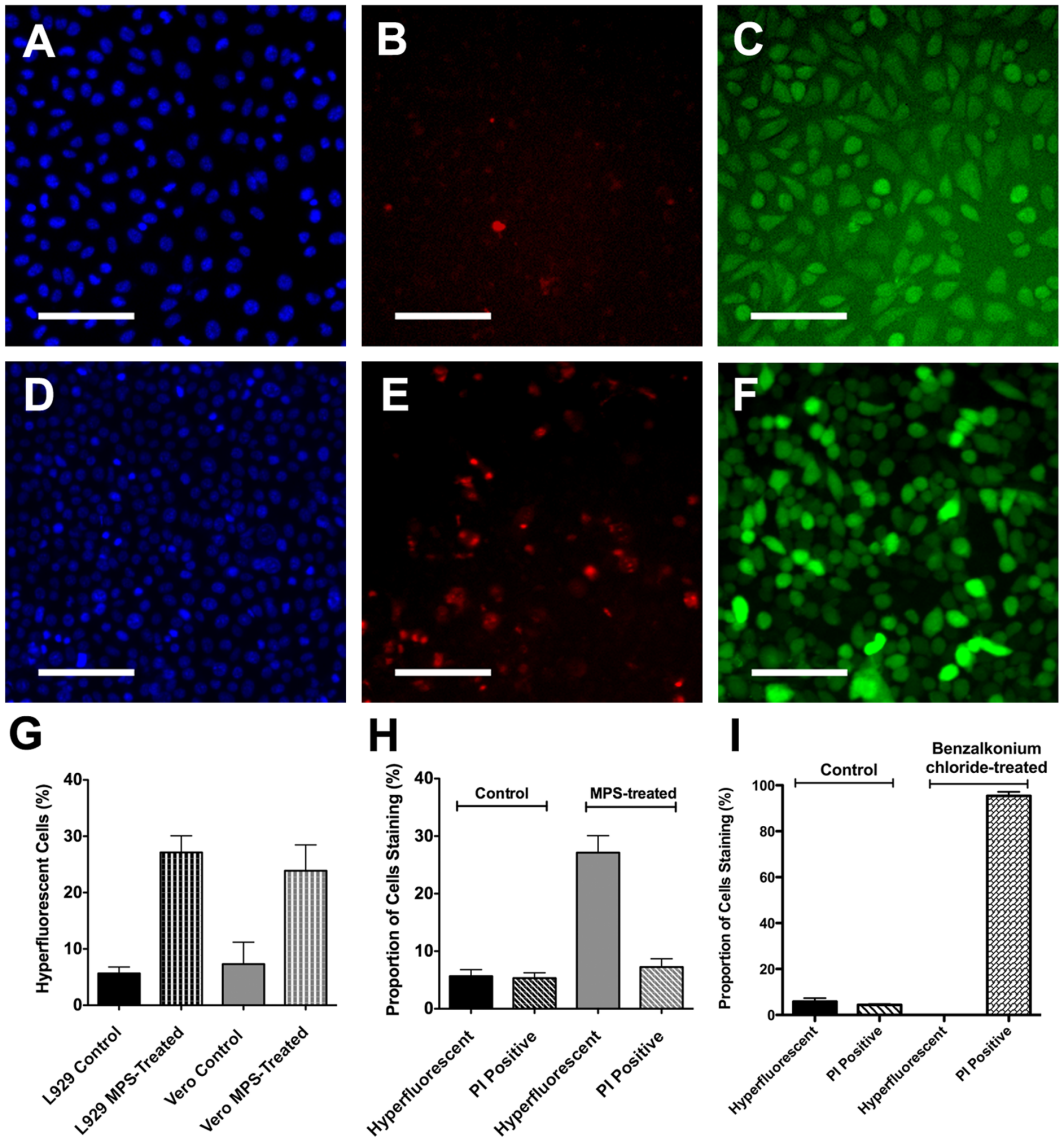
Fluorescein and propidium iodine staining of cells treated with MPS or benzalkonium chloride. L929 cultures were treated with growth medium containing 25% ReNu MultiPlus® MPS or PBS (control) and stained with Hoescht 33342, propidium iodide and fluorescein following an overnight incubation. Typical appearance of control cells is shown after Hoescht 33342 staining (A), propidium iodide staining (B) and fluorescein staining (C), with the equivalents for MPS-treated cells shown in (D) to (F). No correlation was evident between propidium iodide-staining cells and fluorescein hyperfluorescent cells. Notably the number of hyperfluorescent cells was significantly increased after MPS-treatment for both L929 (n = 20 wells) and Vero cultures (n = 6 wells) (p≤0.01) (G). However the overall numbers of PI-staining cells did not increase in L929 cells (n = 20 wells) (H) (this was not tested for Vero cells). Conversely treatment with benzalkonium chloride dramatically increased the number of PI-positive cells, but resulted in no detectable fluorescein hyperfluorescent cells (I). Data shown are representative of several experiments. Scale bars in (A) to (F) represent 100 µm. Bars in (G) to (I) represent standard error.

**Figure 3:**
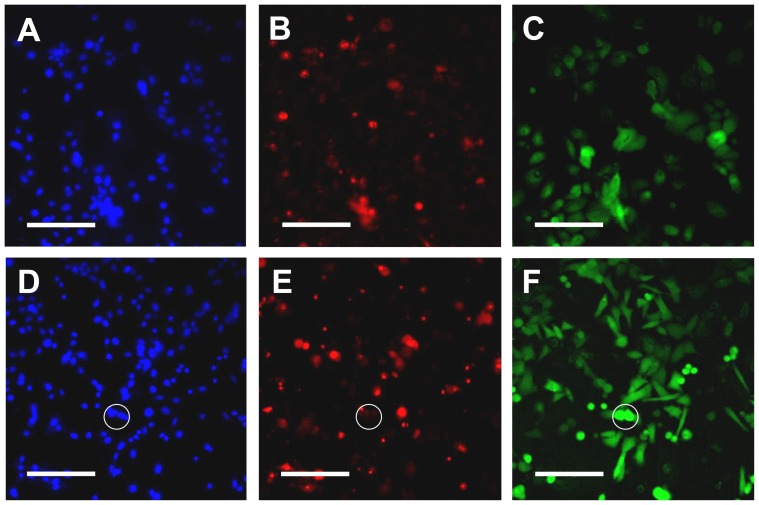
Fluorescein and propidium iodine staining of hCTEpi cells treated with MPS. hTCEpi cultures were treated with hCTEpi growth medium containing 25% ReNu MultiPlus® MPS or PBS (control) and stained with Hoescht 33342, propidium iodide and fluorescein following an overnight incubation. Typical appearance of control cells is shown after Hoescht 33342 staining (A), propidium iodide staining (B) and fluorescein staining (C), with the equivalents for MPS-treated cells shown in (D) to (F) (in all cases n = 6 wells). The numbers of fluorescein hyperfluorescent cells clearly increased after MPS-treatment. Importantly hyperfluorecent cells were not also found to stain strongly with PI (one example is indicated by the circle). Data shown are representative of several experiments. Scale bars in (A) to (F) represent 100 µm.

Cell cultures treated with MPS showed substantially higher proportions of hyperfluorescent cells compared to control culture (L929: 27.1±3.0% vs. 5.7±1.1%; t = 6.8, p<0.001 and Vero: 23.9±4.6% vs. 7.3±3.9%; t = 3.2, p = 0.01), along with small morphological changes (Fig. 2A to Fig. 2G). Conversely exposure of cell cultures to MPS (ReNu MultiPlus®) resulted in an apparent small increase in PI-staining (Fig. 2B and 2E), however when numerically assessed using HCA analysis this trend did not reach significance (control 5.3±0.9% vs. MPS 7.3±1.4%; t = 1.1, p = 0.27) (Fig. 2H). Importantly, microscopic observation revealed that individual hyperfluorescent cells were rarely also stained by PI (Fig. 2E and Fig. 2F), strongly suggesting that fluorescein hyperfluorescence was not directly related to cell death. To confirm and further explore the mechanisms related to this finding, we exposed L929 cultures to toxic concentrations of benzalkonium chloride (a membrane disrupting agent) and measured levels of hyperfluorescence and PI staining. Almost all cells treated with benzalkonium chloride stained with PI (95.4±5.2%), whereas no detectable hyperfluorescent cells were apparent in these dead cultures (Fig. 2I), i.e. there was a complete absence of hyperfluorescence in cells that were deliberately lysed. We additionally confirmed that our generic findings (obtained with an epithelial and fibroblast cell line) also occurred for human corneal epithelial cells. Treatment of hTCEpi cells with MPS also increased numbers of hyperfluorescent cells, and in both control and MPS cultures, these cells did not also stain strongly with PI; conversely cells staining with PI did not show hyperfluorescence (Fig. 3).

### Fluorescein loss and uptake requires active living cells

**Figure 4:**
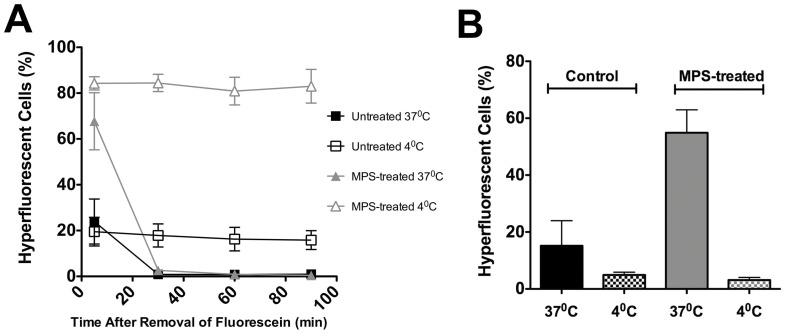
Dependence of cellular loss or entry of fluorescein on cellular activity. The proportion of hyperfluorescent L929 cells is shown after control (n = 12 wells) or MPS (n = 8 wells) treatment, for cells treated at 37°C with fluorescein, and then washed and observed at either 37°C or 4°C. Cultures held at 4°C do not release fluorescein (A). Conversely cells treated with fluorescein at either 37°C or 4°C, prior to observation at 4°C (the latter to prevent further change in fluorescein hyperfluoresence after treatment) are shown; cultures treated at 4°C appear to accumulate much lower levels of fluorescein (n = 4 wells) (B). Data shown are representative of several experiments; bars represent standard deviation and standard error in A and B, respectively.

We next investigated directly whether the extent of cellular activity affected any *loss* of hyperfluorescence after staining, by comparing levels of fluorescein hyperfluorescence over time for cells treated at normal physiological temperatures (37°C), and then washed and observed at either 37°C, or alternatively washed and observed at a low temperature (4°C). We found that fluorescein-treated cells washed in saline at 4°C, and subsequently maintained at 4°C for up to 100 min showed no change in the overall proportion of hyperfluorescent cells. However when washing was carried out at normal physiological temperature and this temperature was utilized for subsequent observation there was a very marked decline in the proportion of cells showing hyperfluorescence (Fig. 4A). Together these data show that internalized fluorescein leaves cells under normal physiological conditions, but when the cells are in a quiescent state induced by low temperature this removal of fluorescein does not occur. Interestingly the overall proportion of hyperfluorescent cells in cultures held at 4°C was higher than that found in our earlier experiments (presumably as in the latter some washes were carried out at 37°C, thereby causing some loss of fluorescein) (Fig. 4A).

We also examined the influence of temperature on the *entry* of fluorescein, by carrying out treatment of cells at either 37°C or 4°C. After exposure to fluorescein at either temperature, we then carried out all subsequent observation at 4°C (in the light of our earlier finding that the proportion of hyperfluorescent cells rapidly declines at 37°C). After initial treatment at 37°C, and five minutes after removal of the 37°C fluorescein stock solution, 54.9±7.0% of MPS-treated cells showed hyperfluorescence, compared with only 15.2±7.6% of control-treated cells. Much lower proportions of hyperfluorescent cells were observed when the initial fluorescein treatment was carried out at 4°C (3.1±0.8% and 5.0±0.8%, respectively) (Fig. 4B). Together, these data strongly suggest fluorescein uptake and release by cells are dependent on active processes within living cells.

## Discussion

In this study we investigated the fundamental cell biology underlying the interaction between fluorescein and fibroblast and epithelial cells in culture. Our initial findings confirmed that fluorescein is able to enter normal sub-confluent cells, as has been suggested previously [14], [19],. Moreover, we found that a sub-population of cells take up appreciably higher levels of fluorescein (‘hyperfluorescent’ cells) and that fluorescein was present throughout the cell, including the cytoplasm. These data confirm the clinical observations of Mokhtarzadeh et al [13], who showed that fluorescein was present within the cytoplasm of corneal epithelial cells in patients exhibiting punctate corneal staining and are in line with the clinical investigations reported by Bandamwar et al [20] who found that rinsing of corneas exhibiting solution induced corneal staining did not result in a reduction of staining indicating that the fluorescein present was not pooling at the corneal surface but instead was “strongly associated with corneal epithelial cells or elements thereof”. It should of course be noted that fluorescein may quench at higher concentrations; although the levels of fluorescein within cells in our experiments appear to be too low for such quenching to occur, this cannot be completely excluded with our data. Therefore the apparent high levels of fluorescein in hyperfluorescent cells would need to be confirmed by other methods to be certain.

Significantly, we found that treatment with the MPS used in this work (a product known to be associated with solution induced corneal staining) causes an increase in the proportion of hyperfluorescent cells. These data support the hypothesis that cellular accumulation of fluorescein may underlie clinical observations of fluorescein staining, at least for SICS. This does not exclude the possibility that other interactions of fluorescein with corneal tissues may also contribute, for example fluorescein pooling or preservative-associated transient hyperfluorescence. Additionally we did not examine the effect of cellular confluency on this, which would be an interesting aspect to explore in future studies.

Notably we did *not* find evidence that MPS-treatment of cells caused intense fluorescein staining on the cell surface, as may have been expected, should preservative interactions with fluorescein be responsible for fluorescein hyperfluorescence as shown by Bright et al [21]. However it is unclear whether the levels of the preservative (PHMB) in our experimental models would reach those likely to be encountered at the cornea, and so further work is needed to establish the role of this potential mechanism *in vivo*.

Importantly, we did not find any association between cell hyperfluorescence and cell death for control and MPS-treated cell populations. We therefore conclude that hyperfluorescence is not a simple cell biological marker for the end-stage of cell death, at least for our two diverse cell types. Furthermore, we found that fluorescein uptake in living cells is dependent on those cells being intact, as cells deliberately lysed by benzalkonium chloride exhibit minimal levels of hyperfluorescence. These data strongly suggest that the hyperfluorescence observable during SICS does not simply reflect increased numbers of dead cells being present, in response to the presence of MPS. Consistent with this is our observation that both fluorescein uptake and release are profoundly influenced by temperature, indicating that these processes involve active transport in living cells. It should be noted that one previous study has reported that the polarization of fluorescein is affected by temperature [22]; however the ArrayScan II system we used in temperature experiments does not make use of polarizing filters, and so it is likely that the changes we measured in numbers of hyperfluorescent cells incubated at different temperatures genuinely reflects cellular changes in fluorescein levels. These data further support our findings that the fluorescein is distributed throughout the cell, is concentrated by living cells, and does not accumulate preferentially in lysed or dead cells. These findings may also be relevant to the use of fluorescein in cell biological research as a marker for other substances, as our data indicate that any inadvertently non-conjugated fluorescein contaminants are likely to be taken up by living cells, potentially introducing artifacts.

Together these data suggest that the increased number of hyperfluorescent cells seen after exposure to MPS reflect an imbalance between active uptake and release processes for fluorescein (i.e. increased entry or decreased exit of fluorescein) occurring in these cells. We cannot however exclude the possibility that adverse events such as apoptosis occur in the hyperfluorescent cells, which would not impact the membrane integrity, but which might change the balance between the rate of fluorescein entry and exit. The finding of poor association of hyperfluorescence with end stage cell death is of interest in interpreting the broader clinical significance of SICS and other forms of fluorescein staining. In conclusion our data suggest that hyperfluorescent ‘punctate’ spots reflect active cells with intact membranes, rather than dead cells with lysed membranes.
